# Heterologous Expression of *CFL1* Confers Flocculating Ability to *Cutaneotrichosporon oleaginosus* Lipid-Rich Cells

**DOI:** 10.3390/jof8121293

**Published:** 2022-12-11

**Authors:** Silvia Donzella, Concetta Compagno

**Affiliations:** Department of Food, Environmental and Nutritional Sciences (DeFENS), University of Milan, Via L. Mangiagalli 25, 20133 Milan, Italy

**Keywords:** lipid production, downstream process, flocculation, *Cutaneotrichosporon oleaginosus*, heterologous expression

## Abstract

Lipid extraction from microbial and microalgae biomass requires the separation of oil-rich cells from the production media. This downstream procedure represents a major bottleneck in biodiesel production, increasing the cost of the final product. Flocculation is a rapid and cheap system for removing solid particles from a suspension. This natural characteristic is displayed by some microorganisms due to the presence of lectin-like proteins (called flocculins/adhesins) in the cell wall. In this work, we showed, for the first time, that the heterologous expression of the adhesin Cfl1p endows the oleaginous species *Cutaneotrichosporon oleaginosus* with the capacity of cell flocculation. We used Helm’s test to demonstrate that the acquisition of this trait allows for reducing the time required for the separation of lipid-rich cells from liquid culture by centrifugation without altering the productivity. This improves the lipid production process remarkably by providing a more efficient downstream.

## 1. Introduction

The increasing use of vegetable oils for non-food applications has led to significant ethical problems associated with the “food vs. fuel” issue. In fact, the demand for biofuels has resulted in a rise in the cost of vegetable oils, from which 90% of biodiesel is derived, and this in turn has been driving the search for alternative oil sources [1,2]. Oleaginous yeasts are included among microorganisms known for their ability to accumulate lipids [2,3,4]. Yeast SCO (single-cell oil) shows a fatty acid profile very similar to vegetable oils [5], making this microbial product attractive for biodiesel production. Moreover, SCO could also be suitable for animal and human nutrition due to its high content of unsaturated fatty acids [6]. Oleaginous yeasts can accumulate intracellular lipids up to 70% of their dry weight (DW) and are considered “easy” for industrial production since they are fast-growing and do not require land usage, thus allowing oil production that is unaffected by geographical, seasonal, and climate limitations and does not compete with any agricultural activity [7,8,9]. Furthermore, they can utilize low-value substrates as well as wastes [4,10,11]. Lipid accumulation occurs on sugar-rich media in concomitance with a shortage of other nutrients, usually nitrogen, when sugar concentration is still high [2,3].

In order to implement the industrial use of SCO, it is necessary to make the bioprocess sustainable and cost-effective [12]. Koutinas et al. [13] reported that the oil cost of using the oleaginous yeast *Rhodosporidium toruloides* would be 5.5 US $/kg. At present, one of the critical points that limits microbial as well as microalgae oil production processes is the separation of oil-rich cells from liquid fermentation media and lipid extraction, which makes the downstream for lipid recovery problematic on a large-scale [14]. Lipids are synthesized intracellularly, and their extraction is carried out after cell disintegration, an energy-intensive process requiring the drying/dewatering of the biomass, which makes the overall process costly [14,15,16]. Lipids are extracted from dry biomass usually by using solvents or by applying mechanical pressure to squeeze out the oil from the broken cells, but this method is relatively slow and requires large amounts of biomass [17,18].

Centrifugal separation is a common approach for harvesting microorganisms, but it consumes a large amount of energy [19]. A rapid and cheap system by which particles can be removed from a suspension is flocculation. This method represents a suitable option for recovering cells from liquid media and processing large volumes of cultures at a minimal cost. Flocculation by employing physical, chemical, or biological means has been regarded as an efficient method for harvesting algal biomass [20,21,22]. The bio-flocculant molecule MBF2-1 produced by *Paecilomyces* was used to flocculate the yeast *Trichosporon fermentans* and remove further organic materials from the fermented soybean oil refinery wastewater [23]. However, some inorganic/organic flocculants may be toxic to the environment, and residues can contaminate cells or fermentation products [24]. In some yeast species, flocculation is a natural way for cells to adhere to each other or on surfaces [25]. This ability is based on important properties, such as invasive growth and sexual reproduction [26,27]. Cell–cell adhesion is also a mechanism for cells in a solution to escape from harsh conditions by sedimentation, thus enhancing their survival. This phenomenon underlies biofilm formation, which increases cell survival under stress conditions [28,29]. By forming multicellular agglomerates, flocculation can protect the inner cells from multiple stresses, including the presence of antimicrobial compounds and ethanol.

The natural phenomenon of yeast flocculation is due to the lectin-mediated adhesion of adjacent cells [25,26,27,28,29,30]. Lectin-associated proteins, called flocculins (Flo proteins), localized on the cell surface of one cell, are able to bind to mannose residues in the cell wall of adjacent cells, thereby producing yeast flocs that may contain thousands of cells. In *Saccharomyces cerevisiae*, flocculins are anchored to the cell wall by a glycosyl-phosphatidyl-inositol (GPI) anchor [31,32], and their structure consists of three domains. Flo proteins interact with other yeast cells through their N-terminal mannose-binding domain [33,34]. Genes encoding for flocculins contain tandem repeats, and their number determines not only the strength of flocculation but to some extent the sugar specificity of the flocculation proteins as well [31].

Yeast flocculation is considered a useful technology for decreasing the cost of bioethanol production [35]. Yeast flocs easily sediment at the bottom of the fermentation vessel, reducing the need to use costly methods to remove cells from the medium, such as centrifugation or chemical flocculation [36,37]. In the case of the fermentation of toxic lignocellulose hydrolysates, flocculation has been shown to increase tolerance to inhibitors, resulting in faster fermentation [38].

Cfl1p was the first adhesin identified in *Basidiomycota*, and the corresponding gene is one of the most induced during *Cryptococcus* sexual development [39,40]. This protein is cell-wall-associated, but it is also released [41,42]. The released form works as a signal regulating colony morphology in autocrine and paracrine ways [40]. Cfl1p does not contain the common domain present in the known Ascomycete adhesins, the GPI anchor, as well as an N-terminal carbohydrate or peptide binding domain (it also does not contain the middle domain, which contains serine/threonine-rich repeats) [25,42]. The domain organization of Cfl1p shows the presence of an N-terminal EGF motif and an amylogenic region, which are predicted to cause cell–cell adhesion [40]. The C-terminal region, named the SIGC domain (signal C-terminal domain), is highly conserved among Cfl1p homologs in different *Basidiomycetes* species [40]. Overexpression of the *CFL1* gene in *Cryptococcus* has been shown to confer a wrinkled colony morphology and an enhanced ability to flocculate [43].

In this work, for the first time, we demonstrate the possibility to obtain a flocculating oleaginous yeast by the heterologous expression of *CFL1* in *C. oleaginosus*. Among the oleaginous species, *C. oleaginosus* has been described as one of the most promising for the sustainable conversion of a wide range of agro-industrial wastes to new-generation oils [44]. Its ability to grow on various carbon sources as well as accumulate a high content of lipids, and its good tolerance to the main growth inhibitors make *C. oleaginosus* a good candidate for industrial application [45,46]. Its strong ability to flocculate allows for separating the recombinant lipid-rich cells from the liquid medium at the end of the lipid production process in a more efficient way in comparison to the wild-type strain.

## 2. Materials and Methods

### 2.1. Strains and Growth Conditions

Yeast strains used in this work: *Cutaneotrichosporon oleaginosus* ATCC 20509 and the obtained transformant strains. *Escherichia coli* strain used for cloning and plasmid propagation was One Shot^®^TOP10.

For long-term storage, yeast strains were maintained at −80 °C on 15% (*v/v*) glycerol.

YPD medium containing 10 g/L yeast extract (Biolife, Milan, Italy), 20 g/L peptone (Biolife, Milan, Italy), and 20 g/L glucose (Sigma Aldrich, Milan, Italy).

YNB medium containing Yeast Nitrogen Base without amino acids, ammonium sulfate 0.17 g/L, (Difco BD, Milan, Italy), ammonium-sulfate 5 g/L, and 0.1 M MES hydrate (4-Morpholineethanesulfonic from Sigma Aldrich, Milan, Italy) to maintain a pH of 6. In plates, 15 g/L of agar (Sigma Aldrich, Milan, Italy) was added to the same medium.

Medium B (high C/N ratio): glucose 50 g/L, (NH_4_)_2_SO_4_ 1 g/L, KH_2_PO_4_ 1 g/L, MgSO_4_7H_2_O 0.05 g/L, NaCl 0.01 g/L, CaCl_2_ 0.01 g/L, yeast extract 1 g/L, and 0.1 M MES (2-(N-morpholino) ethanesulfonic acid) buffer/KOH to maintain a pH of 6.

All media were sterilized in an autoclave (Cavallo S.r.l., Milan, Italy) at 7.3 psi (0.5 atm) and at 112 °C for 30 min.

Pre-cultures were prepared by inoculating cells from glycerol stocks and cultivating on YPD in bluffed flasks with an air-liquid ratio of 5:1 at 28 °C in a rotary shaker at 150 rpm overnight. After completing this part of the process, cells were harvested by centrifugation (5000 rpm/2300 rcf, 10 min in Eppendorf 5415D centrifuge) and inoculated at OD_660_ 0.1 in bluffed flasks. Cell growth was monitored by measuring the increase of optical density at 660 nm (OD_660_) using a spectrophotometer (Eppendorf, Milan, Italy).

### 2.2. Assembly of cfl1PT_hy Vector

All primers employed for plasmid assembly are listed in Table S1 (Supplementary Material). Molecular biology reagents were purchased from ThermoFisher, Milan, Italy. Phusion High-Fidelity DNA Polimerase was used for PCR product amplification, and restriction enzymes were used for vector assembly. NucleoSpin^®^Plasmid, NucleoSnap^®^ Plasmid Midi, and PCR clean-up were purchased by Macherey-Nagel.

The pTU57_hygr plasmid containing the construct for the resistance to hygromycin B (optimized synthetic gene) was placed under the control of the constitutive promoter of the gene that codes for aldolase and its terminator, which was synthesized by BaseClear B.V. For the assembly of the expression system for *C. oleaginosus*, the expression cassette pTU-PT, used in our previous work and composed by *Rhodosporidiobolus azoricus* (previously known as *R. azoricum*) PGK promoter and *Ashbya gossypii* TEF1 terminator, was employed [47].

The *CFL1* sequence (from NCBI database, Gene ID: 2353120) encoding for Cell flocculin 1 (Cfl1p) from *Cryptococcus neoformans* was synthesized by BaseClear B.V. The synthetic gene was amplified from pUC57-Cfl1 plasmid (primers ForCfl1 and RevCfl1, Table S1), digested with NdeI and cloned in pTU-PT vector, obtaining pTU-PT-Cfl1 plasmid.

To allow the subsequent excision of the promoter-cfl1-terminator construct, two restriction sites (HindIII and BcuI) were inserted at the ends by PCR with specific primers (cfl1PT_BcuI_for and cfl1PT_HindIII_rev, Table S1). The PCR product was purified and digested with the restriction enzymes BcuI and HindIII.

**Figure 1:**
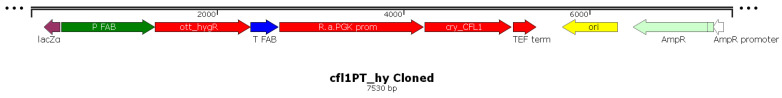
Structure of linearized plasmid for Cfl1 expression.

The same restriction enzymes were used to digest the vector pTU57_hygr, which was synthesized by BaseClear B.V. Thus, the insertion of the cfl1PT construct into the pTU57_hygr plasmid could only take place in the correct orientation (Figure 1).

The ligation reaction with which the competent strain of *E. coli* DH5α was transformed was thus set up. The plasmid obtained (cfl1PT_hy, Figure 1) was thus extracted and sent for sequencing to check for the absence of errors by using SEQ4, SEQ5, CECK3, and CECK2 primers (Table S1).

Once the correctness of the construct was verified, the final plasmid cfl1PT_hy was treated with the BamHI enzyme to excise the cassette (linearized) that needed to be transferred into yeast (5740 bp).

### 2.3. Transformation Protocol

After overnight growth in 100 mL of YPD, the OD of the cultures was verified to be under 10 OD. The broth was transferred into 50 mL tubes and centrifuged at 5000 rpm at room temperature for 10 min. The pellet was resuspended in 10 mL of 0.1 M Lithium acetate and centrifuged. The pellet was washed again with 1 mL of 0.1 M Lithium acetate, transferred into a 2 mL tube, and incubated in Lithium acetate that was stirred for 1 h at 25 °C. To eliminate all traces of Lithium acetate, the cells were centrifuged at 3500 rpm for 5 min and washed in cold 1 M sucrose solution at least twice. The cells were then resuspended in an adequate volume of sucrose to obtain a 10^8^ cells/mL solution. This resuspension was aliquoted in 100 μL per treatment and stored on ice. DNA was added (resuspended in MilliQ water) at a maximum volume of 10 μL. Cells and DNA were transferred to a sterile and cold 0.2 mm electroporation cuvette (VWR International, Radnor, PA, USA), and electroporation was performed under the following conditions: 2.5 KVolts, 50 μF, and 100 Ω for 4–5 ms (BIORAD Gene Pulser II and BIORAD Controller Plus). After each electroporation, fresh YPD medium was added to the resuspension to recover all the cells to be placed in 15 mL tubes. The treated cells were incubated for at least 4 h at 30 °C at 150 rpm. The resuspension was then centrifuged at 3500 rpm for 5–10 min and the supernatant was removed. Cells were resuspended in 100 μL of fresh YPD and plated on YPD with hygromycin (50 μg/mL) as a selection marker.

These were placed at 30 °C for about 5 days until there were single colonies of sufficient size to be collected. The clones obtained were streaked again on YPD + 50 μg/mL hygromycin and subsequently inoculated in liquid culture (YPD + 50 μg/mL hygromycin), and the obtained material was used for the extraction of genomic DNA.

### 2.4. DNA Extraction

To isolate genomic DNA, pellets of 30 OD_660_ cells were resuspended in 0.5 mL of 0.05 M Tris–HCl/0.02 M EDTA at a pH of 7.5. This suspension was transferred to a precooled tube with an equal volume of glass beats (425–600 µm). Mechanical lysis was performed using a TissueLyser LT alternating 2 min of agitation at 50 Hertz with 1 min in ice for 4 cycles. The supernatant was added with 25 μL of SDS 20% (*w/v*) and processed according to Querol et al. [48].

### 2.5. RNA Extraction

To isolate genomic DNA, pellets of 30 OD_660_ cells were collected, quickly frozen in liquid nitrogen, and stored at −80 °C until use. Total mRNA was then extracted using Presto^TM^ Mini RNA Yeast Kit (RBY050, Geneaid Biotech Ltd., New Taipei City, Taiwan) and treated with DNase I (Sigma-Aldrich, Milan, Italy) to remove genomic DNA. The quality of the extracted RNA was verified by running it on a gel and reading it at 260/280 nm. Moreover, cDNA was synthesized from mRNA using QuantiTect Reverse Transcription Kit (Qiagen Italia, Milano, Italy). 

### 2.6. Sugar and Nitrogen Determination

The concentrations of glucose during fermentation processes were determined by employing the commercial enzymatic kit (K-GLUHK, Megazyme, Wicklow, Ireland). All the assays were performed in triplicate and standard deviations varied between 1 and 5%. Inorganic nitrogen was determined by employing a commercial enzymatic kit (10542946035, R-Biopharm AG, Pfungstadt, Germany).

### 2.7. Dry Weight Determination and Lipid Quantification

Cells were collected from the medium (2 mL of cell culture) by centrifugation (10 min at 13,200 rpm/16,100 rcf in Eppendrof 5415D centrifuge). The pellets were dried overnight at 105 °C.

Lipid content was determined via the sulfo-phospho-vanilline colorimetric method (Spinreact, Girona, Spain) on the washed cell pellets corresponding to approximately 30 OD_660_, suspended in 0.5 mL of cold redistilled water. The assays were performed in triplicate and standard deviations varied between 1 and 5%.

### 2.8. Microscopy Imaging

Cells were examined using an Axio Imager microscope (Zeiss, Carl Zeiss Inc., Thornwood, NY, USA).

### 2.9. Flocculation Test

A simplified Helm’s method was used to determine the flocculation ability [49,50]. After determining the OD_660_ of the sample to be analyzed, the cells are diluted in a cuvette to a density of 0.5 OD, shaken, and read with a spectrophotometer at 660 nm (A1). They are then left motionless for 30 min or 2 h and read back on the spectrophotometer (A2). A decrease in cell concentration is therefore observed in the samples that tend to flocculate more. The flocculation percentage is obtained from the formula [1 − (A2/A1)] × 100 [50]. To compare percentages derived from different experiments, these values were expressed as fold change with respect to the control (ATCC 20509 wild-type strain).

To evaluate the flocculation also in undiluted samples, this protocol was modified by leaving the undiluted cells immobile for 3 h and then measuring the absorbance of the upper part of the culture.

### 2.10. Centrifugation Tests

The culture broth of wild type and transformants was harvested after 90 h of process on medium B, transferred into a 50 mL Falcon tube, and centrifuged at 4000 rpm for 1, 2, 5, and 10 min. Subsequently, the OD_660_ of the supernatants was measured; the consistency of the pellets and the clarity of the supernatants were evaluated by taking pictures.

## 3. Results and Discussion

### 3.1. Engineering C. oleaginosus by Heterologous Expression of CFL1

Strong flocculations of recombinant strains have been proven difficult to achieve by overexpressing *S. cerevisiae FLO* genes because they contain long sequences of internal repeats that can lead to recombination events [31]. In addition, the mannose content in the *C. oleaginosus* cell wall is significantly lower than in *S. cerevisiae* [44], and this could negatively affect the mechanism of flocculation by the heterologous expression of *S. cerevisiae* flocculins [29]. For these reasons, we shifted our search toward adhesins expressed in the phylum *Basidiomycota,* to which *C. oleaginosus* belongs. Cfl1p is an adhesion protein that promotes flocculation and biofilm formation in *C. neoformans* [43]. Interestingly, it was found that the overexpression of *CFL1* resulted in an attenuation of virulence, indicating that Cfl1-mediated cell adhesion negatively modulates virulence [43]. *C. oleaginosus* has been already engineered for the production of modified fatty acids [51], and for the over-accumulation of triglycerides by introducing pyruvate dehydrogenase bypass genes (PDH) [52]. To introduce the genome of C. oleaginosus to the heterologous gene CFL1 and express it strongly and constitutively, we constructed a recombinant cassette that contained the promoter of the *R. azoricus* gene encoding for phosphoglycerate kinase (PGK) and the *A. gossypii* TEF terminator (Figure 1). The cassette also contained the promoter and the terminator of *R. azoricus* fructose aldolase (FBA) for the expression of the *hygR* gene, used as a selective marker (Figure 1). After integrating the *C. oleaginosus* genome, we obtained clones on plates containing 50 μg/mL hygromycin, indicating that the *R. azoricus* promoter and terminator worked properly in the *C. oleaginosus* background.

**Figure 2:**
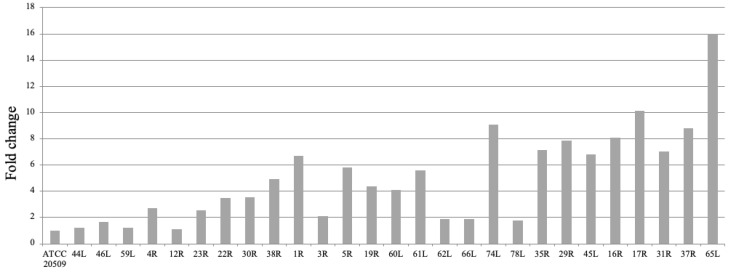
Screening of recombinant clones by Helm’s test. The percentage of flocculation is expressed as fold change in comparison to the control strain (wild-type ATCC 20509), which is set to 1.

Most of the clones exhibited a wrinkled morphology, as reported for a recombinant *C. neoformans* strain over-expressing *CFL1* [45]. The Helm’s test [49,50] was utilized to evaluate the flocculation capacity in 27 recombinant clones that were cultivated on YPD for 24 h. A wide range of flocculation was displayed (Figure 2).

Few clones behaved like the control strain, and the others flocculated in a range that was 2 to 16 times greater than the control. A heterogeneous phenotype could be expected due to different causes. Random integration of the gene cassette into the genome can affect the expression of *CFL1* and can cause other flocculation side effects. Further integration of multiple copies of the gene can result in a stronger expression and then, in turn, can determine a more marked phenotype. On the other hand, as observed for other engineered strains of unconventional yeasts [53], the analysis of the integration locus often provides no conclusive information, because this locus could encode proteins that have unknown effects. This problem will be overcome when strategies for homologous integrations into specific loci become available.

Eight clones that showed the greater flocculation capacity by Helm’s test were selected and genetically analyzed in order to confirm the integration and the expression of the gene cassette encoding Cfl1p (Figures S1 and S2).

**Figure 3:**
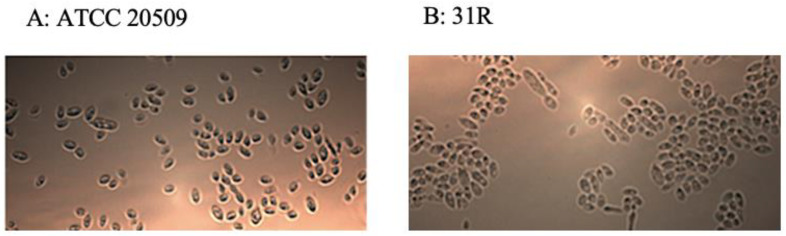
(**A**): Wild-type cells (ATCC 20509). (**B**): Recombinant 31R cells forming aggregates.

Pictures taken during microscope examinations showed that during the growth, the recombinant cells expressing *CFL1* formed aggregates (Figure 3B) in comparison to the control that grew in an unbundled way (Figure 3A).

### 3.2. Flocculation of CFL1-Recombinant Strains Containing High Amount of Lipid

The locus effect caused by the random integration of the cassette containing *CFL1* into the genome can also affect lipid production. To assess this aspect, the selected recombinant strains were cultivated on mediums at a high C/N ratio (a condition that triggers lipid accumulation).

**Figure 4:**
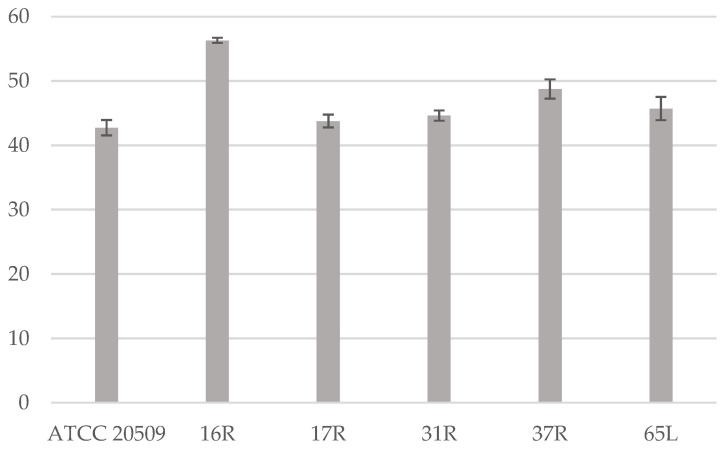
Lipid content (expressed in % of dry weight) produced by the recombinant strains after 65 h of growth.

The 5 selected clones grew faster and showed similar or higher lipid content than the control strain, ranging from 42% to 58% of DW (Figure 4).

**Figure 5:**
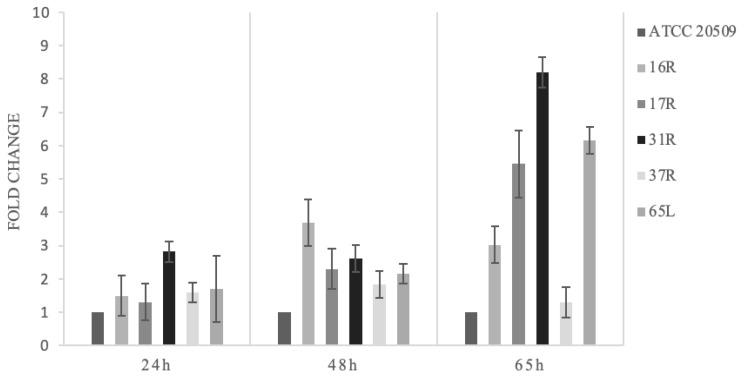
Helm’s test after 24, 48, and 65 h of growth in B medium. The percentage of flocculation is expressed as fold change in comparison to the control strain (wild-type ATCC 20509), which is set to 1.

To characterize the flocculation ability of these strains under suitable conditions for the process of lipid production, the Helm’s test was repeated on samples collected at different times during cultivation on mediums at a high C/N ratio. However, cultivation under lipogenic conditions resulted in a different pattern of flocculation in comparison to the one observed on YPD (see Figure 2 and Figure 5 for comparison). After 24 h, all the strains maintained higher fold changes in comparison to the control (but lower than on YPD). Under lipogenic conditions, the best-performing strain was 16R, exhibiting a 3.8 times greater flocculation (on YPD it was 8 times greater). Nevertheless, after 65 h, the flocculation pattern changed, and the strongest trait (8-fold greater than the control strain) was displayed in strain 31R (Figure 5).

**Figure 6:**
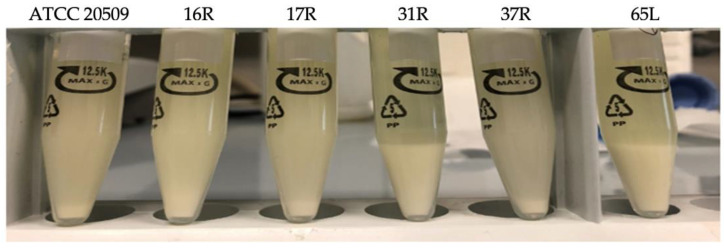
Undiluted cultures collected after 65 h of growth and rested for 3 h.

A high level of intracellular lipids causes cells to float. However, given the lipid content of the recombinant strains (Figure 4) and their flocculating ability (Figure 5), it is possible to note that after 65 h of cultivation, even the strain 16R (the one that accumulated the highest amount of lipids (58% of DW)) exhibited a capacity to form a pellet that was 3 times stronger than the control strain’s capacity to form a pellet. These results were also achieved by comparing the cell pellets that were obtained by leaving the samples for 3 h without any stirring (Figure 6). This further confirmed that the presence of Cfl1p determines the flocculating trait in cells that are able to accumulate a very high lipid content.

**Figure 7:**
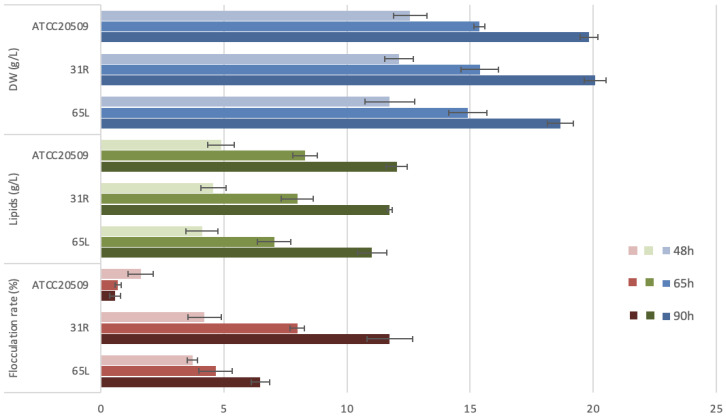
Time course of dry weight (DW, g/L), lipid accumulation (g/L), and flocculation rate (%) of the wild type, 31R and 65L. The flocculation rate was calculated using the Helm’s test and expressed in % (see Materials and Methods).

The performances of the two best recombinant strains (31R and 65L) were analyzed in detail during the lipid production process by collecting samples at different times and comparing them with the wild-type strain’s dry weight, lipid concentration, and flocculation rate (Figure 7). In terms of biomass and lipid production, both recombinant strains showed no significant differences with the wild type, highlighting the fact that the expression of *CFL1* did not affect biomass and lipid synthesis. On the contrary, a completely different flocculation rate trend was detected: during lipid accumulation, the wild type decreased its flocculation capacity, but both 31R and 65L increased their flocculation rate up to 13-fold.

**Figure 8:**
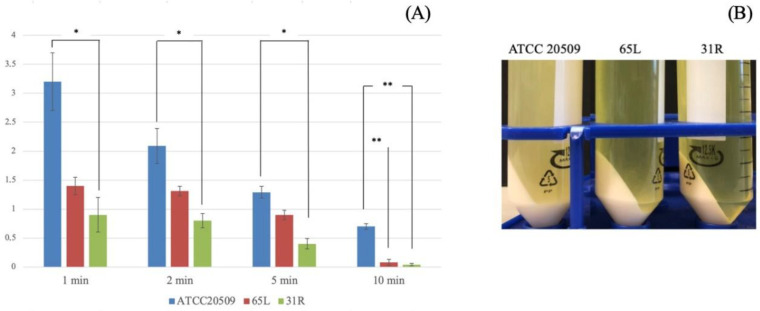
(**A**) OD_660_ of the upper part after centrifugation for different times at 4000 rpm. *: *p* < 0.05. **: *p* < 0.01. (**B**) Pellets of cultures collected after 90 h of process on medium B and centrifuged for 10 min at 4000 rpm.

To verify if the flocculating trait can be used to reduce the cost of separating cells from the medium in fermentation processes for lipid production, we tested the performance of recombinant strains by using centrifugation as a cell-collecting method at the end of the process (after 90 h) (Figure 8). By evaluating the turbidity (OD) of supernatants obtained after different times/durations of centrifugation, we found lower values for both transformed strains in comparison with the wild type, even after only one minute of centrifugation (Figure 8A). Proceeding for longer times, the strain 31R showed the strongest ability to flocculate, allowing a more efficient separation from the fermentation medium due to a consistent reduction of the centrifugation time (Figure 8A,B).

This trait can represent an important aspect in the optimization of the industrial lipid production process since the downstream step heavily contributes to the cost of the overall bioprocess. The cell disruption process, including lipid extraction from cells of oleaginous microorganisms, makes downstream processing problematic for lipid recovery on a large scale, and its cost is considered a major bottleneck for producing biodiesel on a large scale. Different strategies for helping oleaginous species to improve this aspect have been reported [54,55,56]. To avoid the centrifugation of large culture volumes, decantation at a low temperature is considered successful for biomass separation [57]. Flocculation can reduce the decantation time and has been exploited in some industries, such as wine and beer production, to limit the need for time-consuming and expensive cell removal methods, such as centrifugation or filtration. Researchers have focused on various methods to improve the flocculation of the employed yeasts species. In the search for genes conferring strong flocculating traits, the overexpression of *FLO* genes in *S. cerevisiae* has been investigated [58]. The overexpression of *FLO1* was used to obtain a genetically engineered flocculating *S. cerevisiae* strain with good fermentation performance for industrial ethanol production [59]. Wine strains that exhibit flocculant behavior at the end of the wine fermentation process were identified by using high-throughput sedimentation rate assays [60]. In another study, searching for new flocculation genes resulted in the discovery of a chimeric gene that endowed an *S. cerevisiae* strain with a strong flocculation capacity [61]. This trait has also been applied to obtain strongly adherent cells, reducing the potential for cell leakage from the matrix in immobilized systems [62,63].

Flocculation is affected by several fermentation parameters such as pH. The pH can alter the charge of the cell surface and ionization of the flocculin functional groups, modifying its conformation [64]. Factors that decrease the negative electrostatic charges on the cell walls and that increase the cell-surface hydrophobicity cause stronger flocculation, probably by facilitating cell–cell contact [64,65]. Another important aspect that needs to be considered in fermentation processes is the hydrodynamic condition. Stirring the culture could break up cell aggregates and hence must be carefully controlled in order to promote cell collision. In addition, the concentration of suspended cells is also involved in determining the number of collisions needed to form flocs [66]. Usually, at the end of the fermentation process, conditions are more beneficial for the formation of large flocs, because yeast concentration is maximal [66].

## 4. Conclusions

In conclusion, this work demonstrates that the heterologous expression of *CFL1*, encoding a *Cryptococcus* adhesin, endows *C. oleaginosus* with the ability to flocculate under conditions of lipid accumulation. The acquisition of this trait allows for improving the downstream process for the separation of lipid-rich cells from the liquid medium at the end of the lipid production process. In this way, by significantly reducing the efficiency and the time required for culture centrifugation, a critical point that limits the microbial-oil production process can be overcome.

## Data Availability

Not applicable.

## Supplementary Materials

The following supporting information can be downloaded at: https://www.mdpi.com/article/10.3390/jof8121293/s1. Figure S1: Agarose gel of PCR products generated by using Cfl1_for and Cfl1_rev specific primers on DNA of clones obtained by ATCC20509 transformation using cfl1PT_hy plasmid. M: marker, ctr+: cfl1PT_hy plasmid; Figure S2: Agarose gel of PCR products generated by using Cfl1_for and Cfl1_rev specific primers on cDNA (synthetized from mRNA) of clones obtained by ATCC20509 transformation using cfl1PT_hy plasmid. M: marker, ctr+: cfl1PT_hy plasmid; Table S1: Primer sequences employed for cfl1PT_hy assembly and mutant screening.
