# Supplementary material for: Heterologous Expression of CFL1 Confers Flocculating Ability to Cutaneotrichosporon oleaginosus Lipid-Rich Cells
Source: J Fungi (Basel). 2022 Dec 11;8(12):1293. doi: 10.3390/jof8121293 (PMC9786196; doi:10.3390/jof8121293)
Supplement: Supplementary file 1 [file jof-08-01293-s001.zip › jof-2056904-supplementary.pdf]

Figure S1: Agarose gel of PCR products generated by using Cfl1_for and Cfl1_rev specific primers on DNA of clones obtained by ATCC20509 transformation using cfl1PT_hy plasmid. M: marker, ctr+: cfl1PT_hy plasmid.

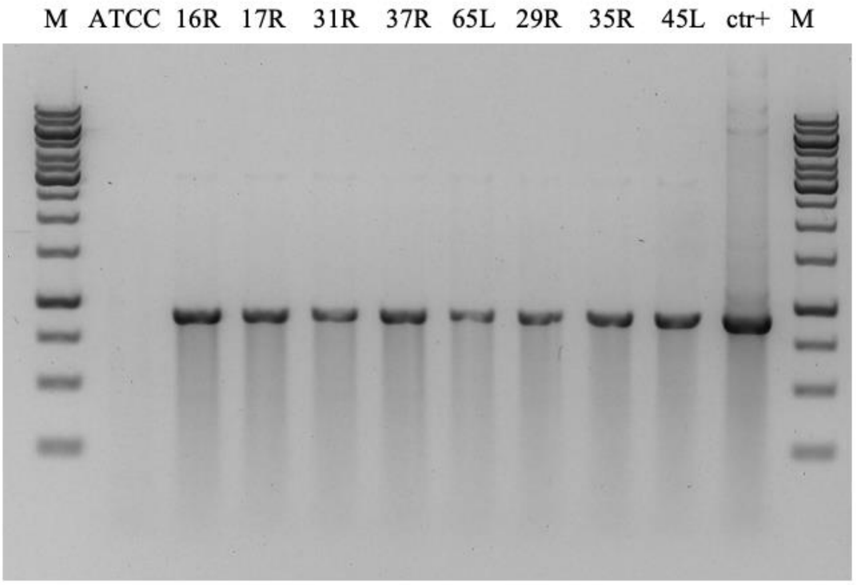

Figure S2: Agarose gel of PCR products generated by using Cfl1_for and Cfl1_rev specific primers on cDNA (synthetized from mRNA) of clones obtained by ATCC20509 transformation using cfl1PT_hy plasmid. M: marker, ctr+: cfl1PT_hy plasmid.

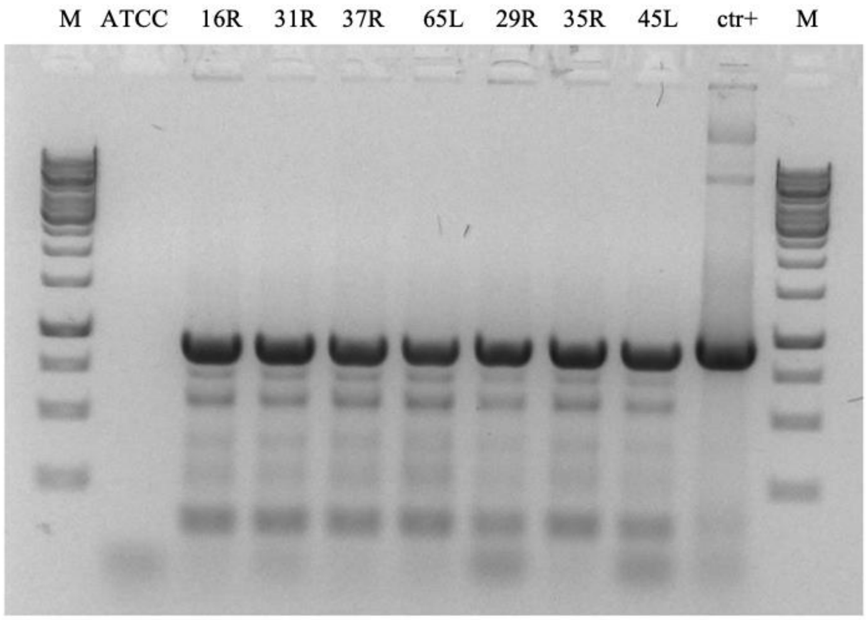

Table S1: Primer sequences employed for cfl1PT_hy assembly and mutant screening.

Primer	Sequence 5'-3'
hygr2_for	ATGAAGAAGCCCGAGCTCACCG
hygr2_ rev	CGCTATCCGCGAGGACCTCG
Cfl1PT_HindIII_rev	tattAAGCTTCGCCCTCGATCAAGC
Cfl1PT_BcuI_for	atgtACTAGTTTGCGGCCGCTTTTCGAC
cfl1_for	gctcgttaccaacatctttgc
cfl1_rev	cactgggtgccattgagacg
SEQ3	CGCTCAAGTACTCCCTG
SEQ4	GGTTGGTGGTATTCCGC
SEQ5	GGCAGATGATGTCGAGG
CECK2	CCGAAGGTGTCGTTGGGTGCGCCG
